# Limited survivability of unbalanced progeny of carriers of a unique t(4;19)(p15.32;p13.3): a study in multiple generations

**DOI:** 10.1186/s13039-017-0330-8

**Published:** 2017-08-04

**Authors:** Darinka Šumanović-Glamuzina, Bernarda Lozić, Piotr S. Iwanowski, Tatijana Zemunik, Zeljka Bilinovac, Beata Stasiewicz-Jarocka, Barbara Panasiuk, Alina T. Midro

**Affiliations:** 1 Department of Pediatrics, University Hospital Mostar, Mostar, Bosnia and Herzegovina; 2 Department of Pediatrics, University Hospital Centre Split, Split, Croatia; 3 Department of Clinical Genetics, Medical University of Bialystok, Waszyngtona St. 13, PO Box 22, 15-089 Białystok, Poland; 4 Department of Medical Biology, School of Medicine Split, University of Split, Split, Croatia

**Keywords:** Estimation of recurrence probability, Miscarriages, Monosomy 4p15.32 → pter, Morphological phenotype, Stillbirth, Trisomy 19p13.3 → pter, t(4;19)(p15.32;p13.3), Wolf-Hirschhorn syndrome

## Abstract

**Background:**

Carriership of a reciprocal chromosomal translocation (RCT) involving the short arm of chromosome 4 (4p) may result in birth of a child with Wolf-Hirschhorn syndrome (WHS) due to monosomy 4p, a priori modified by the impact of the partner chromosome imbalance. Familial transmission studies of RCT enable obtaining empirical risk figures that are essential for genetic counseling. In this study, pedigree data from carriers of a unique t(4;19)(p15.32;p13.3), ascertained by two children with WHS phenotype, were collected through five generations and empirical risk for different pregnancy outcomes was assessed. In addition, the phenotype-karyotype correlation was studied in two unbalanced children against the phenotypes of children (literature data) with pure monosomy 4p15.32 → pter and pure trisomy 19p13.3 → pter, accordingly. The phenotype analysis was conducted using the catalogue of traits according to the Munich Dysmorphology Database. Pedigree segregation analysis was conducted by the direct method according to Stengel- Rutkowski et al.

**Results:**

A double segment imbalance, trisomy 19p13.3 → pter with monosomy 4p15.32 → pter, was diagnosed in WHS progeny at birth. No essential modification of WHS phenotype by the additional trisomy 19p was observed, except for a limited survivability (death in infancy). Pedigree segregation analysis covered 39 relatives showed the probability rate for liveborn with unbalanced karyotype of 3.7 ± 3.6% (1/27), for stillbirth/neonatal death at 7.4 ± 5.0% (2/27), for miscarriage at 22.2 ± 8.0% (6/27), for the chance of having a baby without unbalanced karyotype was estimated at 66.7 ± 9.1% (18/27). In addition, the value of 7.4% for genetic counseling for any carrier of RCT at risk for single segment 19p13.3 → pter imbalance at birth was evaluated as such value have not been estimated so far.

**Conclusion:**

Carriership of a t(4;19)(p15.32;p13.3) is at low risk for an unbalanced child at birth and for stillbirth/neonatal death but high for miscarriages. The chance of having a baby without unbalanced karyotype was estimated to be high. Monosomy 4p15.32 → pter together with trisomy 19p13.3 → pter as a double segment imbalance in children with WHS may be connected with a limited survivability in infancy.

**Electronic supplementary material:**

The online version of this article (doi:10.1186/s13039-017-0330-8) contains supplementary material, which is available to authorized users.

## Background

Partial chromosome 4p monosomy results in a well recognizable morphological and behavioral phenotype, known as Wolf-Hirschhorn Syndrome (WHS) (MIM: #194190). That phenotype is defined by characteristic somatic, neurological and behavioral features including prenatal/postnatal growth retardation, microcephaly, seizures, muscular hypotonia, facial dysmorphism, skeletal anomalies, congenital heart defects, antibody deficiencies, urinary tract malformations, and others. The facial gestalt includes prominent and broad nose ridge continuing to forehead, prominent glabella, ocular hypertelorism, epicanthus, highly arched eyebrows, short philtrum, downturned mouth, micrognathia, and poorly formed ears with pits/tags [1–4].

Apart from the essential underlying chromosome aberration, monosomy 4p, WHS may also be due to a double segment imbalance with monosomy 4p included, resulting from meiotic malsegregation of a parental reciprocal chromosome translocation (RCT). In such cases the imbalance of the partner chromosome segment may impact the phenotype, so the clinical diagnosis of WHS may be “biased” by that effect [2, 5].

In this paper we present two children with WHS due to monosomy 4p15.32 → pter together with trisomy 19p13.3 → pter. To our knowledge only one report of WHS due to unbalanced t(4;19) is available in literature, from a family study including a maternal carrier of a subtle t(4;19)(p16.3;p13.3) ascertained by a girl with WHS [6]. In addition, clinical data on pure chromosome 19p trisomy are scanty, too, due to its rarity. To date, only two children with pure 19p13.3 **→** pter trisomy have been reported [7, 8].

Following this study of familial transmission of the unique translocation t(4;19)(p15.32;p13.3) across five generations including follow-up of families with different pregnancy outcomes we are able to propose the empirical risk figures for genetic counseling. In addition, thanks to extensive pedigree data collected, we propose the use of obtained figures for prediction of probability rates for 19p13.3 → pter single segment imbalance in progeny of any carrier of RCT involving that segment.

### Clinical report

#### Child 1

Our proband (V:2, Fig. 1a-d, Additional file 1: Table S1) was a boy born at 39th week of gestation as the second child of young (mother/father: 24/30 years old) and healthy parents (IV:2; IV:3). During pregnancy intrauterine growth retardation (IUGR) was observed. His birth weight was 1880 g (<3rd ct.), length 45 cm (<3rd ct.), and head circumference 29 cm (<3rd ct.). Apgar scores were 8 and 9 points. As a newborn he was small for gestational age and he was transferred to an intensive care unit (ICU) where he developed respiratory distress syndrome, early sepsis, meningitis, renal failure with mineral imbalance, glucose intolerance, seizures, and muscular hypotonia. Feeding difficulties and failure to thrive were observed in course of further hospitalization. Because of his facial gestalt suggestive of WHS, echocardiography, abdominal and cerebral ultrasonography, and clinical laboratory analysis were performed shortly after birth looking for malformations and other clinical abnormalities. Heart defect (atrial septal defect, ASD), mild-brain ventriculomegaly and agenesis of left kidney were found. He was hospitalized several times during infancy due to prolonged seizures. He died at the age of 12 months in course of severe bronchopneumonia, renal failure and drug resistant epileptic status.

**Fig. 1:**
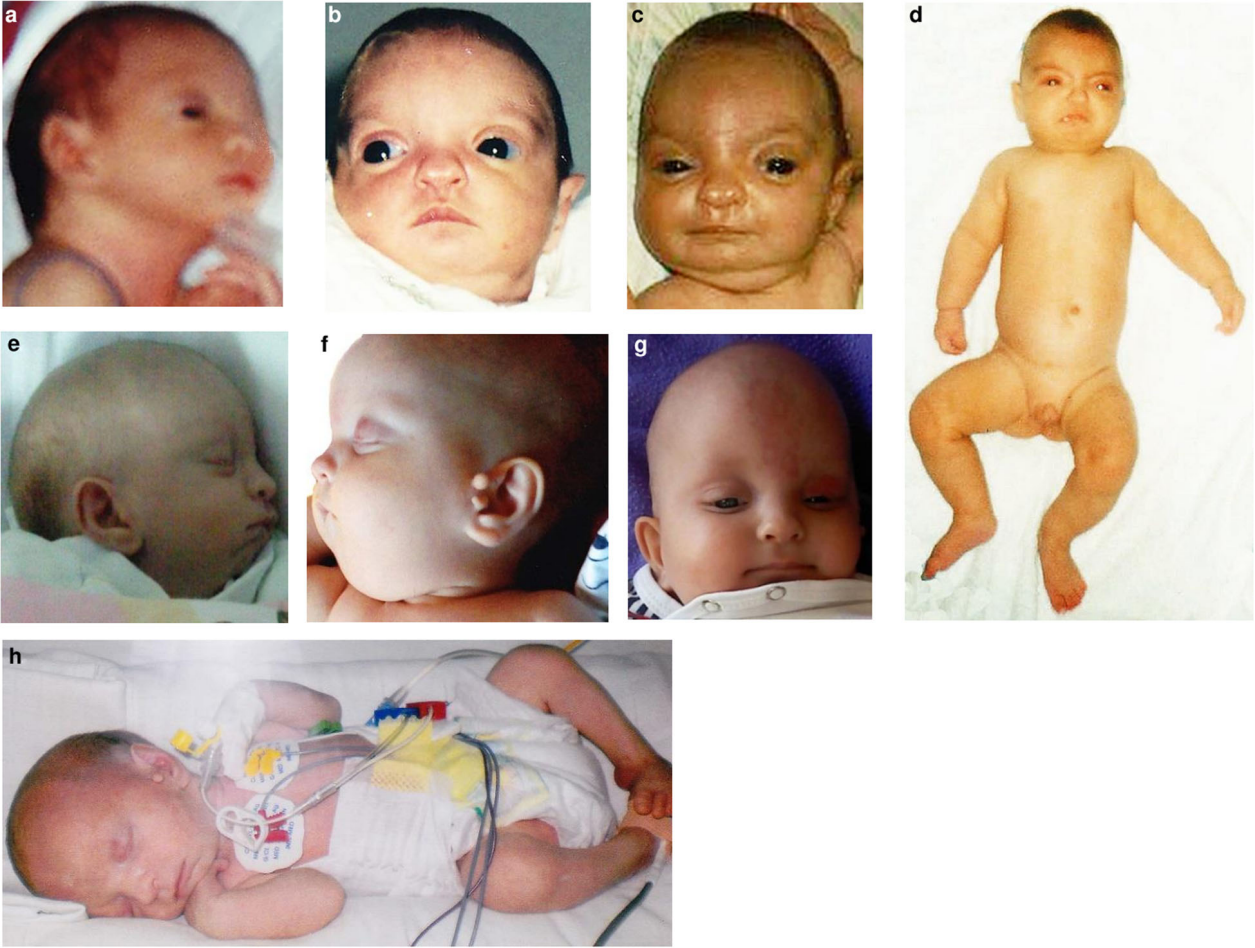
Phenotype of children: Child 1 (V:2) at the age of: **a** 7 days. **b** 40 days. **c** 3 months. **d** 10 months. Child 2 (V:10) at the age of: **e** 20 days. **f**,**g** 3 months. **h** 7 days

#### Child 2

A boy (V:10 Fig. 1e-h, Additional file 1: Table S1) was born at 39th week of third pregnancy of young parents (mother/father: 34/36 years old) (IV: 7; IV: 8). His birth weight was 1690 g (<3rd ct.), length 34 cm (<3rd ct.) and head circumference (<3.ct.). Apgar scores were 9 and 10 points. WHS was diagnosed based on his morphological and clinical features. He was treated several times at a pediatric department due to congenital abnormalities, seizures and severe anemia. Radiology examinations showed heart defect (ASD, pulmonary stenosis and persistent ductus arteriosus), agenesis of left kidney, right side inguinal hernia, and left hip dislocation. He died at the age of 11 months during an episode of bronchopneumonia and persistent seizures.

## Methods

### Morphologic and clinical phenotype analysis

A detailed analysis was performed using the catalogue of morphologic and clinical traits by the Munich Dysmorphology Database (MDDB) [9]. Its outcome was used for comparison with the phenotypes of three children with pure monosomy 4p15.32 → pter at corresponding age published by us earlier [2] and to literature data on phenotypes of two children with pure trisomy 19p13.3 → pter [7, 8]. Facial trait characteristics were supplemented by re-examination of photographs according to the catalogue of traits by MDDB.

### Cytogenetic studies

Cytogenetic analysis was performed on chromosomes from lymphocyte cultures of peripheral blood according to standard procedures. GTG and RBG banding techniques were used. FISH studies using WHSCR probe (4p16.3) (D4S 166-D4S3327) and the 19pter subtelomere specific probe (Cytocell) were done for confirmation of cytogenetic diagnosis and to differentiate terminal and subterminal breakpoint positions on derivative chromosome 19. The interpretation of breakpoint positions of RCT was done according to the ISCN 2013 nomenclature [10].

### Probability rate estimation for different pregnancy outcomes

#### Analysis of meiotic malsegregation of parental chromosomes and prediction of survivality till birth of progeny

Different forms of possible chromosome imbalance following meiotic disjunction and segregation were considered for the meiotic quadrivalent of t(4;19)(p15.32;p13.3). Prediction of survivality till birth was done by empirical data from the pedigree analyzed in conjunction with the published data [2, 11–17].

#### Direct method for pedigree segregation analysis

Pedigree segregation analysis was performed according to Stengel-Rutkowski et al. (1988) [16]. Empirical probability rates were calculated separately for each category of pregnancy outcomes: liveborn with unbalanced karyotype, stillbirth, miscarriage, and child without unbalanced karyotype- further referred to as child with normal phenotype. Ascertainment correction (i. e., elimination of the proband, their parents and relatives in direct line of ascent) was made as per Stene and Stengel-Rutkowski (1988) [18]. Each unfavorable pregnancy outcomes following ascertainment correction was counted and compared to the total number of pregnancies.

The following formulas were used:

$$ p=\frac{a}{n}\pm S $$ $$ S=\sqrt{\frac{a\left(n-a\right)}{n^3}} $$ and when a = 0 $$ {p}_{\max }=1-{e}^{-\frac{1}{2n}} $$.

p: probability (risk) value for different pregnancy outcomes.

a: number of unfavorable pregnancies after ascertainment correction.

n: number of all pregnancies after ascertainment correction.

S: standard deviation.

p_max_: maximum risk value.

e: 2.71828 (base number for natural logarithms).

## Results

### Phenotype analyses

Analysis of morphological and clinical traits of children 1 and 2 with partial monosomy 4p15.32 → pter and trisomy 19p13.3 → pter is presented in Additional file 1: Table S1, along with traits present in three children with pure monosomy 4p15.32 → pter (own analysis previously published [2]) and two patients with pure trisomy 19p13.3 → pter reported by Andries et al. (2002) and Ishikawa et al. (2013) [7, 8].

### Cytogenetic study

Cytogenetic analysis (University Hospital, Split, Croatia) by GTG technique in child 1 (proband) revealed a male karyotype with derivative chromosome 4. Parental chromosome studies showed a maternal balanced RCT between chromosomes 4 and 19, i.e. 46,XX,t(4;19)(p15.32;p13.3), confirmed by RBG technique (Medical University of Bialystok, Poland) (Fig. 3a). The following karyotype of the proband was concluded: 46,XY,der(4)t(4;19)(p15.32;p13.3)mat. Hence, monosomy 4p15.32 → pter with trisomy 19p13.3 → pter resulted from a 2:2 disjunction and adjacent-1 meiotic segregation.

A FISH study (in V:1 carrier) showed the breakpoint position proximal to WHSCR on der(4) and subterminal on der(19) (Fig. 3b). Therefore this RCT was classified at risk for a double segment imbalance in progeny.

### Family studies (Fig. 2)

The familial t(4;19)(p15.32;p13.3) was ascertained by a newborn with WHS phenotype (proband V:2, child 1) with an unbalanced RCT der(4)t(4;19)(p15.32;p13.3)mat. Subsequently another child with WHS (V:10, child 2) was born to a relative (IV:7,8). The unbalanced karyotype also was found in two stillbirths following amniocentesis (V:6; V:8). In all children and stillbirths with unbalanced karyotype the same form of imbalance was observed, i.e. monosomy 4p15.32 *→* pter with trisomy 19p13.3 *→* pter. Family studies revealed 10 carriers of t(4;19)(p15.32;p13.3) (V:1; IV:3–5,8; III:1,8; II:6,8; I:1 or I:2). Five pregnancies resulted in miscarriage including three in proband’s parents (V:3–5) and two in a cousin of proband’s mother (V:9,11).

**Fig. 2:**
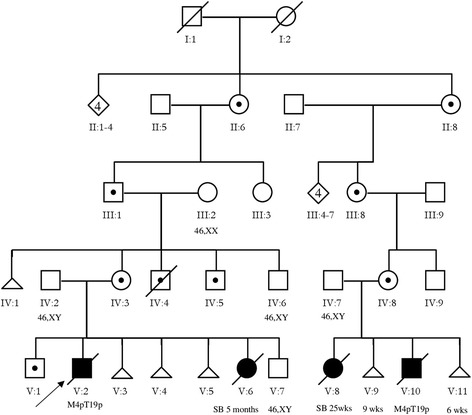
Pedigree data with indication of ascertainment (pedigree was drawn according to Bennett et al. (2008) [34]). Symbols and abbreviations:  proband,  female, male carriers of translocation,  number of siblings,  miscarriage,  dead of child with M4p15.32 → pter/T19p13.3 → pter,  stillbirth

### Recurrence risks estimation for different pregnancy outcomes

#### Analysis of the possible form of imbalance produced by meiotic malsegregation of parental chromosomes in progeny at birth (Fig. 3c, d)

The imbalance in form of monosomy 4p15.32 → pter with trisomy 19p13.3 → pter, resulting from a 2:2 disjunction and adjacent-1 segregation, is compatible with survival, as directly observed in the pedigree.

**Fig. 3:**
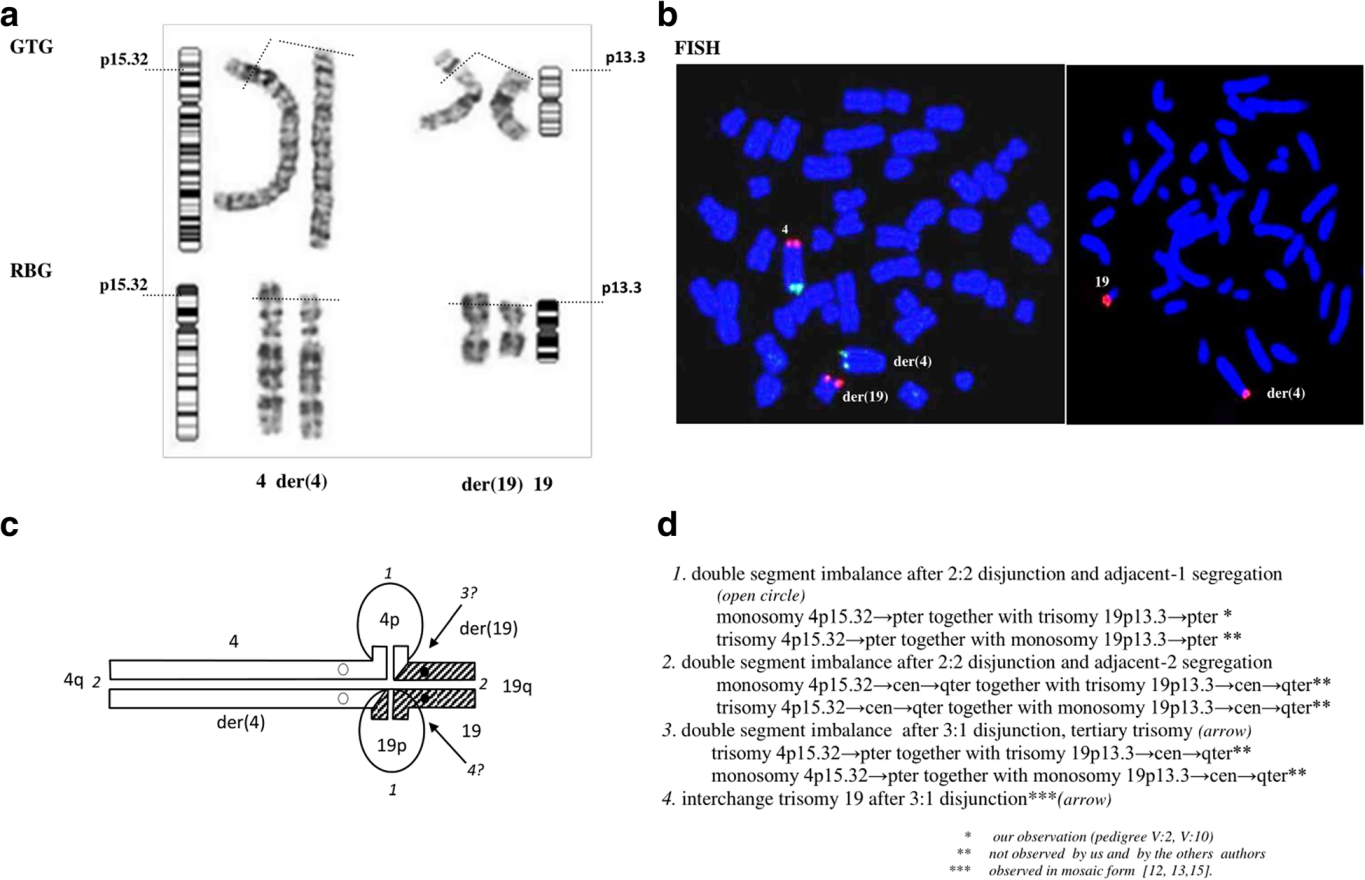
Cytogenetic studies and scheme of meiotic quadrivalent of t(4;19)(p15.32;p13.3). **a** Partial karyotype of t(4;19)(p15.32;p13.3) carrier (V;1) studied by GTG (*upper line*) and RBG (*lower line*) techniques with indication of the breakpoint position on chromosomes and ideograms. **b** FISH studies on metaphase chromosomes of t(4;19)(p15.32;p13.3) carrier (V;1), *left:* WHSCR probe (red) and control probe 4qter (green): one red hybridization signal seen on normal chromosome 4 and second one on der(19)t(4;19)(p15.32;p13.3) indicating the breakpoint position on der(4) proximally to WHSCR; two green signals are seen: one on normal chromosome 4 and second one on der(4)t(4;19)(p15.32;p13.3); *right:* 19pter subtelomere specific probe (red): one hybridization signal is seen on normal chromosome 19 and second one on der(4)t(4;19)(p15.32;p13.3) indicating breakpoint position on der(19) proximally to 19pter subtelomere specific probe. **c** Scheme of meiotic quadrivalent of t(4;19)(p15.32;p13.3) carrier with possible forms of imbalance following meiotic disjunction and segregation. **d** Analysis of survival data in progeny till birth with possible forms of imbalance produced following meiosis in a t(4;19)(p15.32;p13.3) carrier

The alternative product of imbalance in liveborn, trisomy 4p15.32 → pter with monosomy 19p13.3 → pter was not found in that family; however it might be compatible with survival with regards to available reports on children with trisomy 4p15.3, as a part of a double segment imbalance, as well as monosomy 19p13.3 → pter [11, 17].

Progeny with imbalance of longer segments of chromosome 4 (monosomy 4p15.32 → cen → qter or trisomy 4p15.32 → cen → qter) following 2:2 disjunction and adjacent-2 segregation is expectedly lethal and was never observed so far.

Tertiary trisomy 19 or interchange trisomy 19 after 3:1 disjunction were not so far reported in liveborn progeny. However, the survival might be possible for children with trisomy 19 in mosaic form as children with supernumerary r(19) in mosaic forms and pure trisomy 19 were described by others [12, 13, 15].

#### Direct pedigree segregation analysis for different pregnancy outcomes (Table 1)

Following 31 pregnancies of 7 carriers of RCT two children with unbalanced karyotype, two stillbirths with unbalanced karyotype, six miscarriages, and 21 children with normal phenotype were observed. Four individuals, namely the male proband (V:2), his mother (IV:3), grandfather (III;1) and great-grandmother (II;6) were omitted from estimation due to the ascertainment correction. Hence, 27 pregnancies were used for calculation of the probability rates of different categories of pregnancy outcomes.

**Table 1:** Results of a direct segregation analysis of pedigree showing the probability of recurrence estimates obtained separately for: liveborn with unbalanced karyotype, stillbirth/early neonatal death, miscarriage, and child with normal phenotype

Carrier	Parental sex	Liveborn with unbalanced karyotype		Stilbirth		Miscarriage		Child with normal phenotype		Pregnancies		Rate			
		U	u	S	s	M	m	N	n	P	p	u/p	s/p	m/p	n/p
		1	0	1	1	3	3	2	2	7	6	0/6	1/6	3/6	2/6
III:1	PAT	-	-	-	-	1	1	4	3	5	4	−/4	−/4	1/4	3/4
II:6	MAT	-	-	-	-	-	-	2	1	2	1	−/1	−/1	−/1	1/1
IV:8	MAT	1	1	1	1	2	2	-	-	4	4	1/4	1/4	2/4	−/4
III:8	MAT	-	-	-	-	-	-	2	2	2	2	−/2	−/2	−/2	2/2
II:8	MAT	-	-	-	-	-	-	5	5	5	5	−/5	−/5	−/5	5/5
I:1;I:2	MAT? PAT?	-	-			-	-	6	5	6	5	−/5	−/5	−/5	5/5
	Total	2	1	2	2	6	6	21	18	31	27	1/27	2/27	6/27	18/27
									Probability		(%)	3.7±3.6	7.4±5.0	22.2±8.0	66.7±9.1

*U* number of children with unbalanced karyotype, *u* number of children with unbalanced karyotype after ascertainment correction, *S* number of stillbirth, *s* number of stillbirth after ascertainment correction, *M* number of miscarriages, *m* number of miscarriages after ascertainment correction, *N* number of children with normal phenotype, *n* number of children with normal phenotype after ascertainment correction, *P* total number of pregnancies, *p* total number of pregnancies after ascertainment correction

The probability rate for having a liveborn child with unbalanced karyotype was estimated as 1/27, i.e. 3.7±3.6% and classified as low (<5%). The probability rate for a child with normal phenotype was 18/27, i.e. 66.7±9.1% (high probability, >10%). Risk value for stillbirth was 2/27, i.e. 7.4 ±5.0% (medium risk, 5 to 10%) and for miscarriage 6/27, i.e. 22.2 ±8.0% (high risk).

### Probability rate prediction for carriers of RCT at risk for a single segment imbalance of 19p13.3 → pter at birth

No empiric data is available so far for the single segment 19p13.3 → pter imbalance. Therefore on the basis of obtained individual probability rate for imbalance at birth for carriers of t(4;19)(p15.32;p13.3) (direct method) we calculate the probability rate for child with unbalanced karyotype for the single segment 19p13.3 *→* pter imbalance by doubling of value 3.7% obtained in this study according to Stene and Stengel–Rutkowski (1988) [18]. Hence this value is 7.4%.

## Discussion

### Phenotype of monosomy 4p15.32 → pter with trisomy 19p13.3 → pter due to a unique t(4;19)(p15.32;p13.3)

The current report is the second family study on chromosome 19 partnering chromosome 4 in a unique rearrangement resulting in WHS phenotype. A similar RCT t(4;19)(p16.3;p13.3) was first described by Altherr et al. (1991) [6]. In that report, a smaller segment of chromosome 4 was deleted compared to RCT from this report whereas the 19p13.3 → pter imbalance was presumably comparable. Both children reported here had a number of features in common with those reported by Altherr et al. (1991) [6] including IUGR, microcephaly, hyperterlorism, downward eye slant, low set ears, and heart defect, each of those reflecting the WHS phenotype. The following traits were though found in either child reported here but not explicitly reported by Altherr et al. (1991) [6]: weak fetal movements, newborn feeding difficulties and hypotonia, renal agenesis/ectopic kidney with urinary tract anomalies, seizures or abnormal EEG record, cerebral malformation, prominent glabella, hypomimia, long palpebral fissures, laterally descending eyebrows, downturned mouth angles, microgenia, broad ears, broad neck, and inguinal hernia. However, the clinical and morphological report by Altherr et al. (1991) [6] was rather scanty and apparently not based on a systematic analysis of features. Moreover, most of the latter features are compatible with WHS phenotype, too. Therefore no binding conclusion can be drawn which features, if at all, observed in addition in both children with monosomy 4p15.32 → pter with trisomy 19p13.3 → pter are possibly due to a larger 4p deletion.

The phenotype of single segment 4p15.32 → pter monosomy had been delineated by Iwanowski et al. (2011) [2] through a systematic phenotypic analyses based on the same catalogue of traits (MDDB) (see Additional file 1: Table S1). The traits present in both children reported by us but not in patients by Iwanowski et al. (2011) [2] include: reduced birth length, asymmetric skull, high forehead, downward eye slant, short nose ridge, microgenia, inguinal hernia, and urinary tract anomalies. Of them, reduced birth length, high forehead, downward eye slant, short nose ridge, and microgenia were reported in at least one of the only two patients with a pure 19p13.3 → pter trisomy reported so far [7, 8]. It is therefore likely that those latter few features are associated with the 19p trisomy and add to the otherwise dominating features of the WHS (monosomy 4p) phenotype.

Either child reported here showed an early onset of seizures with fatal outcome within the first year of life. We can assume that beside the monosomy 4p15.32 → pter, also the trisomy 19p13.3 → pter contributes to seizure phenotype with an unfavorable outcome. Quigley et al. (2004) [19] who described a RCT t(9;19)(q34.3;p13.3) observed microcephaly, alopecia and autoimmune abnormalities supposedly linked with trisomy 19p. Both our patients died in infancy, had microcephaly and were hospitalized several times because of prolonged seizures and died in status epilepticus after severe bronchopneumonia. Seizures in infancy were also described in cases of microduplication of 19p13.3 region, probably overlapping the trisomic region of both children reported here [8, 20–22]. However, Battaglia et al. (2008) [23] in their study of 87 patients with WHS observed a high percentage (93%) of seizure disorder, generally with a favorable outcome [23]. Shannon et al. (2001) in their WHS study showed that there is relationship between the 4p deletion size, severity phenotype and life expectancy [24]. Although they also observed that survival is poorer in RCTs than in de novo deletions, the difference was not statistically significant.

To date only two patients with pure trisomy of 19p13.3 → pter segment have been reported by Andries et al. (2001) [7] and Ishikawa et al. (2013) [8]. Those reports include multiple malformations i.e. heart defect, developmental delay, and distinctive facial appearance [7, 8]. Other six published reports are on interstitial partial duplications of 19p13.3 [20–22, 25]. There are five further reports of partial trisomy 19p13.3 but with partial monosomy of another chromosome involved (13q, 16p, 9q, 1p) [19, 26–29], in addition to the report on chromosome 4p monosomy discussed above [6]. In all those RCTs, monosomy for the partner chromosome segment most likely contributes to, or even dominates, the ultimate hybrid phenotype.

#### Probability rate estimation for different pregnancy outcomes

In our analysis, the probability rate for birth of a child with unbalanced karyotype, namely monosomy 4p15.32 → pter with trisomy 19p13.3 → pter, was classified as low and it was medium for stillbirth. Those risks are markedly lower compared to a single segment imbalance of RCT at risk for monosomy 4p15.32 → pter at birth estimated at a rate of about 30% [14, 16]. The reason for that difference might be the segment imbalance of the partner chromosome 19 involved in t(4;19). The terminal part of chromosome 19 is known as a gene density region [30]. Our data confirm the general rule saying that the probability rate for viable unbalanced child from carriers of a double segment imbalance is generally lower compared to the rates for carriers of a single segment imbalance [18, 31]. That is because the other forms of imbalance presumably lead to miscarriages. Unfortunately, miscarriage material had not been karyotyped in the families reported.

The analysis of the possible forms of imbalance produced by meiotic malsegregation of parental chromosomes indicates that trisomy 4p15.32 → pter and monosomy 19p13.3 → pter after 2:2 adjacent-1 segregation might also be compatible with survival as either single segment imbalance has been described in liveborn progeny [11, 17]. In addition, survival of children with tertiary trisomy 19 and interchange trisomy could be considered by indirect evidence from published data on mosaic form of trisomy 19 and supernumerary r(19) [12, 13, 15]. Collection of further empirical and cytogenetic data from RCT carrier pedigrees is needed [18, 31].

The frequencies of particular forms of unbalanced gametes produced by carriers of a balanced RCT are contingent on several factors including the size of chromosomal segments involved, their genetic content, sex of RCT carrier, and location of chromosome breakpoints. Survival of progeny with particular imbalances plus possibly other factors influence the risk estimation for the individual RCT carrier [5, 32, 33]. Therefore each empirical study is useful for genetic counseling.

## Conclusions

The evaluation of a phenotype – karyotype correlation did not show any essential modification of WHS phenotype by the additional trisomy 19p13.3 → pter, except for limited survivability. The limited survival (death in infancy of both children reported) is presumably due to additional trisomy 19p13.3 → pter.

Segregation analysis of a large (five-generation) pedigree of t(4;9)(p15.32;p13.3) carriers allowed estimation of probability rates for different pregnancy outcomes for genetic counseling. Carriership of t(4;19)(p15.32;p13.3) is at low risk for an unbalanced child at birth and for stillbirth/neonatal death but high for miscarriages. The chance of having a baby without unbalanced karyotype was estimated to be high. Our pedigree and cytogenetic data collected allowed for calculation, by the indirect method, of a risk value for carriers of RCT at risk for progeny with trisomic, single segment imbalance of 19p13.3 → pter; it is 7.4%.

## Additional file

Additional file 1: Table S1. Phenotype characteristics of two children with monosomy of 4p15.32→pter together with trisomy 19p13.3→pter compared with three children with the pure monosomy 4p15.32 →pter (Iwanowski et al. [2]) and two children with the pure trisomy 19p13.3→pter (Andries et al. [7], Ishikawa et al. [8]). (DOC 177 kb)
