# Supplementary material for: Limited survivability of unbalanced progeny of carriers of a unique t(4;19)(p15.32;p13.3): a study in multiple generations
Source: Mol Cytogenet. 2017 Aug 4;10:29. doi: 10.1186/s13039-017-0330-8 (PMC5545035; doi:10.1186/s13039-017-0330-8)
Supplement: Additional file 1: Table S1. — Phenotype characteristics of two children with monosomy of 4p15.32→pter together with trisomy 19p13.3→pter compared with three children with the pure monosomy 4p15.32 →pter (Iwanowski et al. [2]) and two children with the pure trisomy 19p13.3→pter (Andries et al. [7], Ishikawa et al. [8]). (DOC 177 kb) [file 13039_2017_330_MOESM1_ESM.doc]

**Supplementary materials**

**Supplementary Table S1.** Phenotype characteristics of two children with monosomy of 4p15.32→pter together with trisomy 19p13.3→pter compared with three children with the pure monosomy 4p15.32 →pter (Iwanowski et al., 2011) and two children with the pure trisomy 19p13.3→pter (Andries et al., 2002, Ishikawa et al., 2013).

Note that traits are marked as present based on the original descriptions by Andries et al. and Ishikawa et al., supplemented by own assessments of patient photographs published by those authors with the use of MDDB catalogue of traits (see Methods).

| **Person/reference** | **Iwanowski et al 2011** | | | **Child 1** | **Child 2** | **Andries et al 2002** | **Ishikawa et al 2013** |
| --- | --- | --- | --- | --- | --- | --- | --- |
| **Chromosomal imbalance** | **Single segment imbalance**  **monosomy 4p15.32→pter** | | | **Monosomy 4p15.32→pter and trisomy 19p13.3→pter** | | **Single segment imbalance**  **trisomy 19p13.3→pter** | |
| Sex | f | f | m | m | m | m | f |
| Age at examination [years] | 1 and 1/12 | 1 and 11/12 | 3 and 2/12 | 3/12 | 3/12 | 1 and 9/12 | 3 |
| Weak foetal movements | + | - | - | + | + | ? | ? |
| Preterm delivery (<38 weeks) | + | + | - | - | - | - | + |
| Reduced birth length (<10pc) | - | - | - | + | + | - | + |
| Intrauterine growth retardation (reduced birth weight; <10pc) | + | - | + | + | + | - | + |
| Small birth head circumference (<10pc) | + | + | ? | + | + | ? | + |
| Postpartum feeding/sucking difficulties | + | + | + | + | + | - | + |
| Postpartum apnoea/cyanosis | + | + | - | + | + | - | + |
| Postpartum hypotonia | - | + | + | + | + | - | - |
| Newborn jaundice | + | + | + | + | + | ? | ? |
| Postpartum infection | - | + | - | + | + | - | ? |
| Short stature (<10pc) | + | + | - | + | + | - | + |
| Low weight (<10pc) | + | + | + | + | + | - | + |
| Psychomotor or motor developmental retardation | + | + | + | + | + | + | + |
| Mental retardation | + | + | + | + | + | + | + |
| Large fontanels | - | + | - | + | + | ? | ? |
| Infection proneness | + | + | + | + | + | ? | + |
| Feeding difficulties | + | - | - | + | + | ? | + |
| Intestinal functional disturbance | + | + | + | - | - | ? | ? |
| Heart defect | + | + | - | + | + | ? | + |
| Renal agenesis/  ectopic kidney | + | - | + | + | + | ? | + |
| Urinary tract anomaly | - | - | - | + | + | ? | - |
| Muscular hypotonia | + | + | - | + | + | - | - |
| Seizures or abnormal EEG | + | + | + | + | + | ? | ? |
| Cerebral malformation or structural anomaly | + | + | - | + | + | ? | ? |
| Weak voice | - | + | - | + | + | ? | ? |
| Hearing loss | ? | + | - | - | - | ? | ? |
| Microcephaly (<10pc) | + | + | + | + | + | + | + |
| Asymmetric skull | - | - | - | + | + | - | - |
| High forehead | - | - | - | + | + | + | + |
| Narrow forehead | + | + | - | - | - | - | - |
| Prominent forehead | + | + | + | + | + | ? | + |
| Prominent metopic suture | - | + | + | + | + | - | - |
| Prominent glabella | + | + | + | + | + | - | - |
| Flat glabella | - | - | - | - | - | - | + |
| High frontal hairline | - | + | + | + | - | + | + |
| Diffuse frontal hairline | + | + | - | - | ? | + | + |
| Sunken temporal region | + | + | + | - | - | - | - |
| Visible temporal or facial veins | - | + | - | - | - | ? | - |
| Fine and fair scalp hair /sparse hair | - | + | - | + | + | + | + |
| Round face | - | + | + | + | + | + | + |
| Broad face | + | - | - | + | + | + | + |
| Low midface | - | + | - | + | + | - | - |
| Hypomimia | - | + | - | + | + | ? | ? |
| Full cheeks | + | + | + | + | + | + | + |
| Hypertelorism | + | + | - | + | + | - | + |
| Epicanthal folds | + | + | + | - | + | - | - |
| Upward eye slant | - | + | + | - | - | - | - |
| Downward eye slant | - | - | - | + | + | - | + |
| Broad (long) palpebral fissures | + | - | + | + | + | - | - |
| Narrow (short) palpebral fissures | - | - | - | - | - | - | + |
| Low palpebral fissures or ptosis | + | + | - | - | - | ? | - |
| S-shaped lower lid | + | - | - | + | - | ? | ? |
| Protruding eyes | - | + | - | + | + | - | - |
| Large eyes | + | + | + | + | + | - | - |
| Strabismus | + | + | + | - | - | ? | + |
| Long eyelashes | + | - | - | ? | - | ? | ? |
| Sparse eyelashes | + | + | - | ? | + | ? | ? |
| Curved eyelashes | + | + | + | ? | - | ? | ? |
| Sparse or medially diffuse eyebrows | + | + | - | + | + | ? | + |
| Laterally descending eyebrows | + | - | - | + | + | - | + |
| High-arched eyebrows | + | - | + | + | + | - | + |
| Broad nose root | + | + | + | + | + | + | + |
| Prominent nose root | - | + | - | + | - | ? | ? |
| Long nose ridge | *-* | *+* | *-* | - | - | *-* | *-* |
| Short nose ridge | - | - | - | + | + | + | - |
| Broad nose ridge | + | + | + | + | + | + | - |
| Flat nose ridge | + | - | - | + | - | ? | - |
| Broad nose tip | + | + | + | + | + | + | + |
| Anteverted nostrils | - | - | - | - | - | + | + |
| Downturned nose tip | - | + | - | + | - | - | - |
| Short nasal wings | + | - | + | + | + | + | + |
| Long nasolabial distance | - | - | - | - | - | + | - |
| Short nasolabial distance | - | - | + | + | + | - | + |
| Broad philtrum | + | - | + | + | + | + | - |
| Prominent philtrum columns | + | + | - | + | + | ? | - |
| Philtrum columns continuing into nares | + | + | + | + | + | - | - |
| Downturned mouth angles | + | + | + | + | + | + | - |
| Short mouth fissure/microstomia | - | - | - | - | - | - | + |
| Long mouth fissure/macrostomia | - | - | - | - | - | + | - |
| Bow-shaped mucous upper lip | + | + | - | + | - | - | + |
| Everted/protruding mucous/integumental upper lip | + | + | + | + | - | - | + |
| Inverted/narrow mucous upper lip | - | - | - | - | + | + | - |
| Oligodontia or anodontia | ? | + | - | + | - | ? | ? |
| Cleft palate | + | + | - | - | - | ? | ? |
| Receding integumental lower lip/inverted mucous lower lip | + | - | + | + | + | + | + |
| Microgenia | - | - | - | + | + | - | + |
| Retrogenia | - | + | - | - | - | - | - |
| Abundant submandibular soft tissue | + | - | + | + | + | + | ? |
| Broad ears | + | - | - | + | + | ? | ? |
| Unequal ears | + | + | - | - | - | ? | ? |
| Preauricular pits/tags | + | + | + | - | + | ? | ? |
| Low set ears | - | + | - | + | + | + | + |
| Short helix root | - | + | - | ? | + | ? | ? |
| Poorly rolled descending helix edge | + | + | - | ? | + | ? | - |
| Flat corpus anthelicis | - | - | + | ? | - | ? | - |
| Prominent corpus anthelicis | + | - | - | ? | - | ? | + |
| Flat crus superius anthelicis | + | - | + | ? | + | ? | ? |
| Deep concha | - | + | - | ? | + | ? | ? |
| Long concha | - | + | - | ? | + | ? | ? |
| Prominent or everted antitragus | + | + | - | - | + | ? | ? |
| Short ear lobe | + | + | - | - | - | ? | ? |
| Broad neck | - | + | - | + | + | + | - |
| Scoliosis/  kyphoscoliosis | ? | - | - | - | - | ? | + |
| Hypospadias | ? | ? | + | + | + | - | - |
| Inguinal hernia | - | - | - | + | + | - | - |
| Umbilical hernia | - | - | - | + | - | ? | ? |
| Sacral pit or pilonidal sinus | + | - | + | + | + | ? | ? |
| Thin arms | - | - | - | - | + | - | ? |
| Distally tapering fingers | + | + | + | + | - | ? | ? |
| Hyperconvex fingernails | - | + | - | - | + | ? | ? |
| Left hip dislocation | - | - | - | - | + | - | + |
| Club feet | + | - | - | - | + | - | ? |
| Fibular deviation of big toe | + | + | + | - | + | ? | ? |
| Hyperconvex toenails | + | + | - | - | + | ? | ? |
| Pale skin | - | - | - | + | - | ? | ? |
| Dimples | - | + | + | - | - | ? | ? |

+: trait is present; -: trait is absent; ?: no data or trait not informative
